# Supplementary material for: Far-reaching consequences of trait preferences for animal social network structure and function
Source: Behav Ecol. 2025 Nov 21;37(1):araf132. doi: 10.1093/beheco/araf132 (PMC12784201; doi:10.1093/beheco/araf132)
Supplement: araf132_Supplementary_Data [file araf132_supplementary_data.zip › Version for press, Suppl figs, trait prefs.pdf]

Supplementary figures

Brask *et al.*: Far-reaching consequences of trait preferences for animal social network structure and function

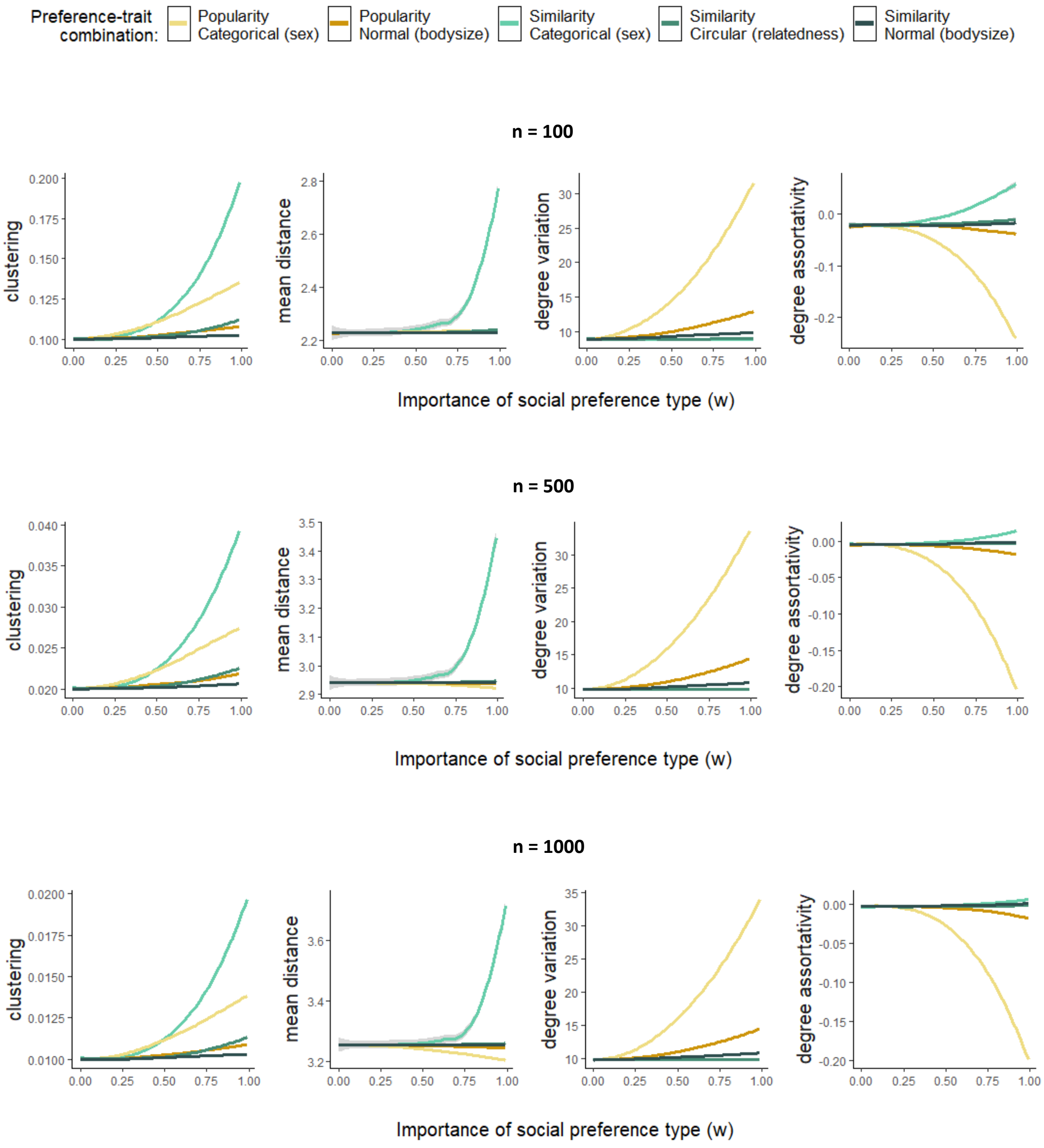

Fig. S1. Effects of trait-based social preferences on social network structure for different network sizes. The change in four network metrics with increased importance of social preferences, for each preference-trait combination (see legend), for different network sizes (given by row headers. Upper row = network size used in main analysis).

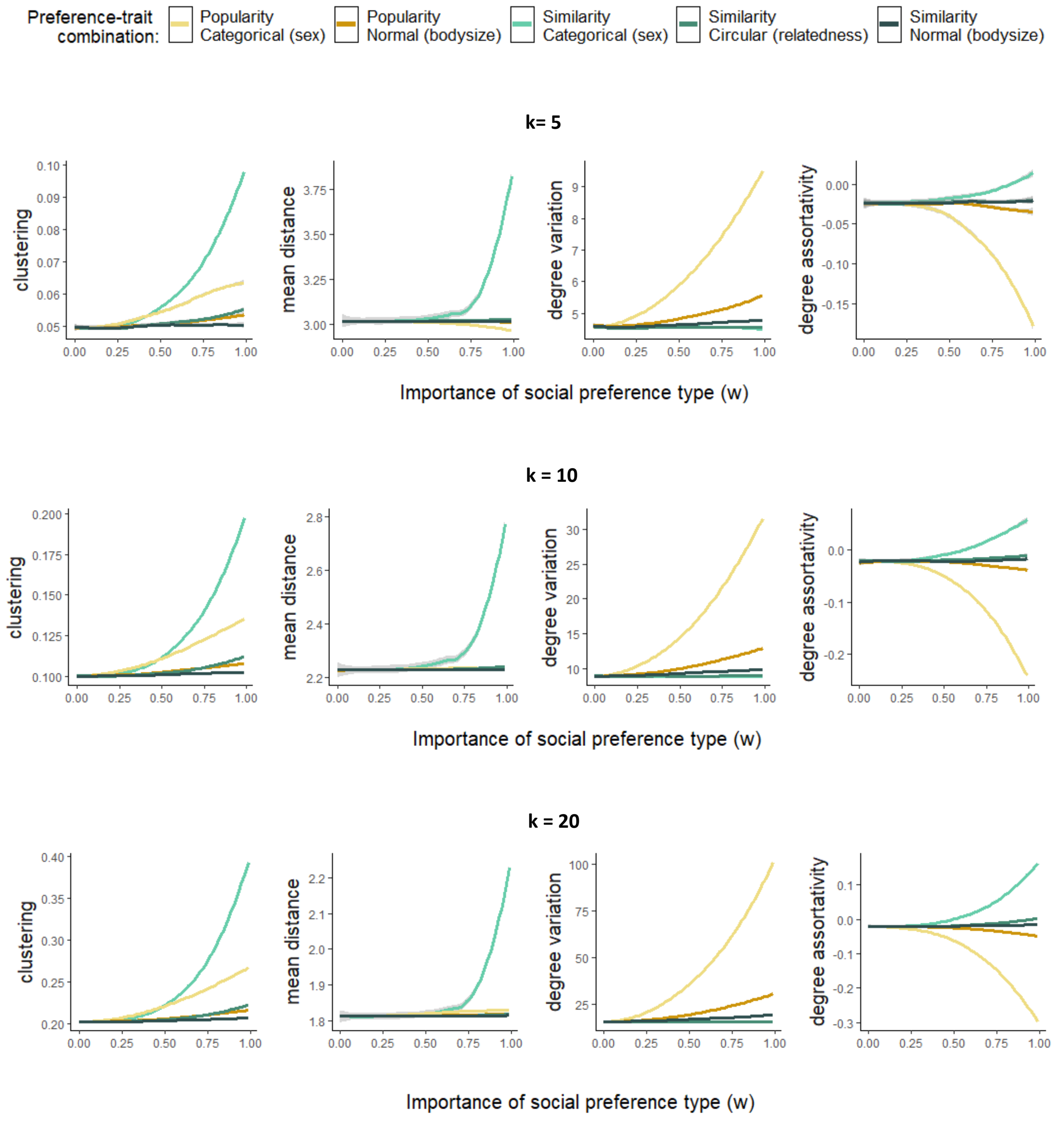

Fig. S2. Effects of trait-based social preferences on social network structure for different average degrees. The change in four network metrics with increased importance of social preferences, for each preference-trait combination (see legend), for different average degrees (given by row headers. Middle row = average degree used in main analysis).

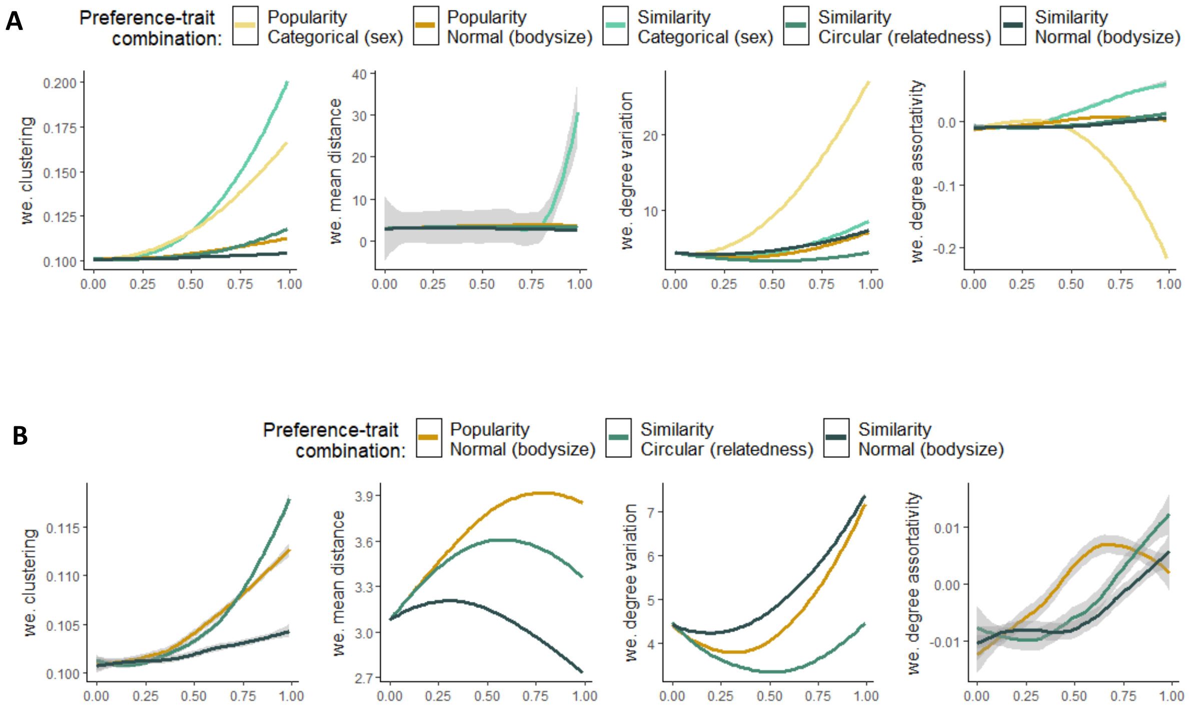

Fig. S3. Effects of trait-based social preferences on social network structure for weighted network metrics. The change in four weighted (we.) network metrics with increased importance of social preferences, for each preference-trait combination (see legend). A: all preference-trait combinations shown. B: only combinations with continuous traits shown.

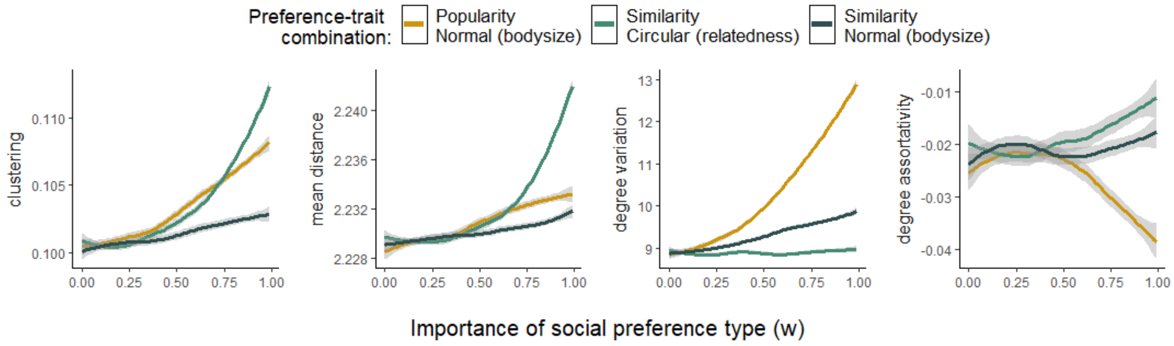

Fig. S4. Effects of trait-based social preferences on social network structure, with only continuous traits shown. The change in four network metrics with increased importance of social preferences, for each of three preference-trait combinations that involve continuous traits (see legend).

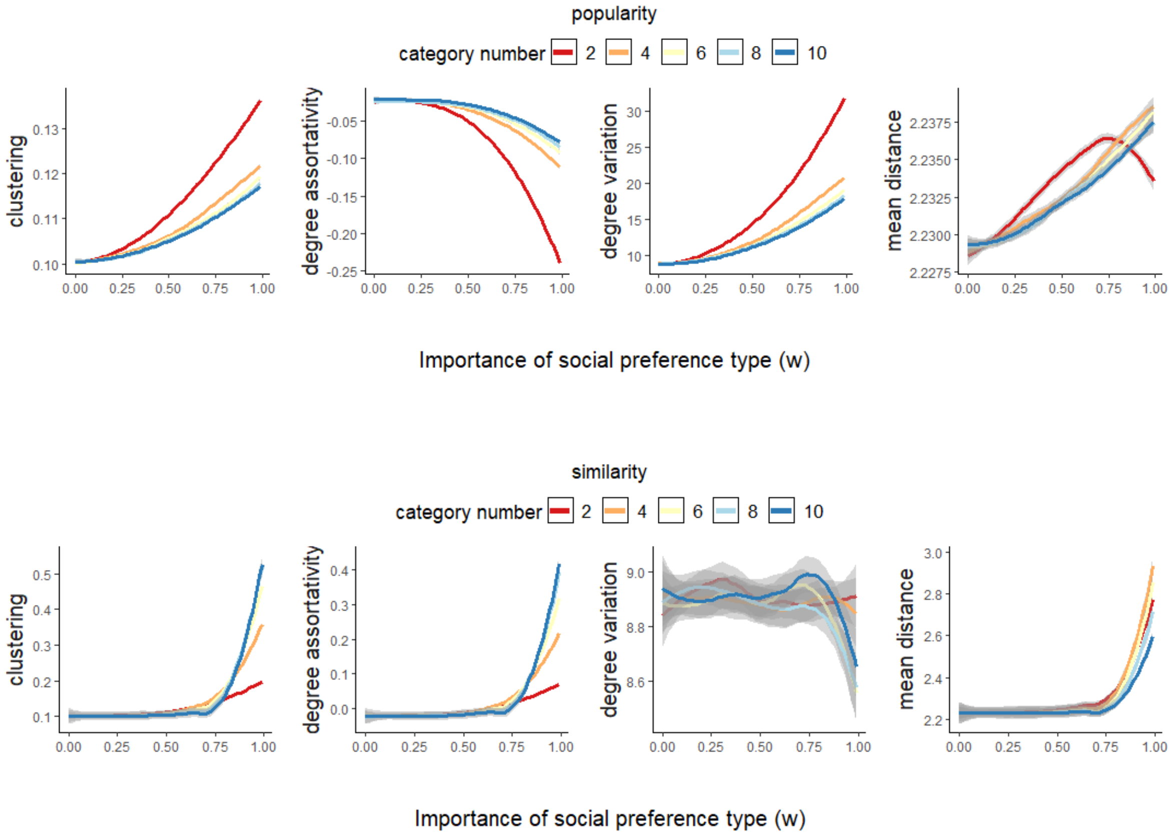

Fig. S5. Effects of trait-based social preferences on social network structure for categorical traits with different numbers of categories. Each line corresponds to the effect of either popularity preferences (upper row of plots) or similarity preferences (lower row of plots), combined with a categorical trait with a different number of categories (see legends).

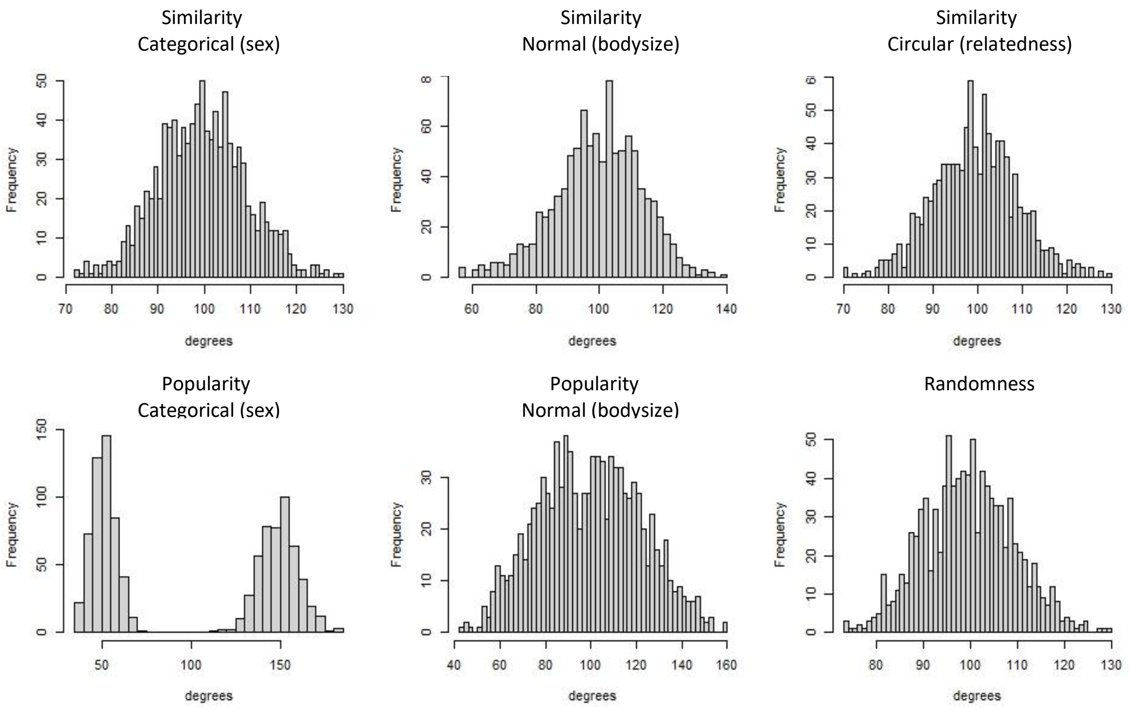

Fig. S6. Degree distributions of networks with trait-based social preferences. Example degree distributions of networks based on the different combinations of preference type and trait type (given by subplot headers) and randomness (no preferences).

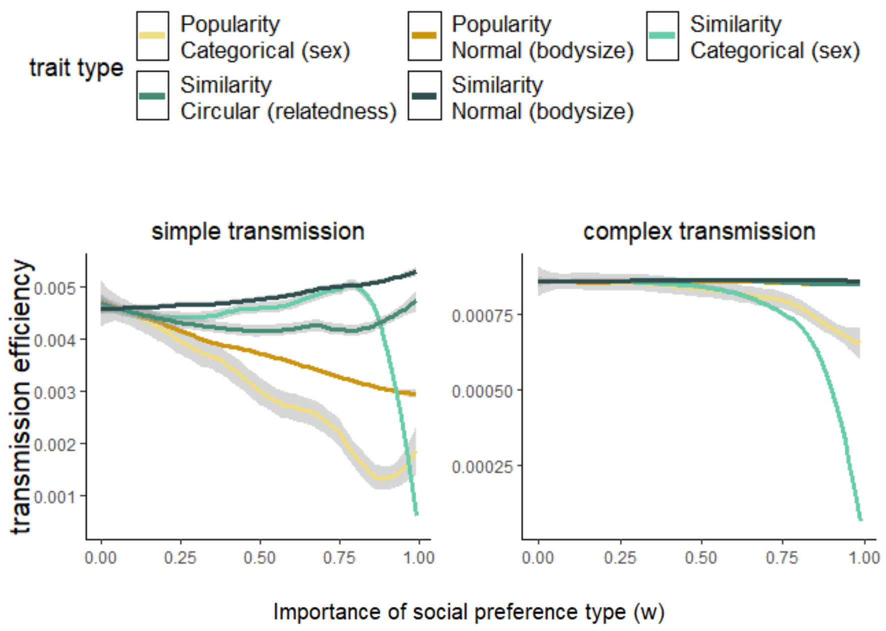

Fig. S7. Effects of trait-based social preferences on social network transmission, where transmission depends on edge weights. The change in transmission efficiency with increased importance of social preferences, for each preference-trait combination (see legend) and two types of transmission (given by subplot headers), for the case where the probability of transmission between two individuals depends not only on the presence of an edge between them, but also on the weight of the edge.

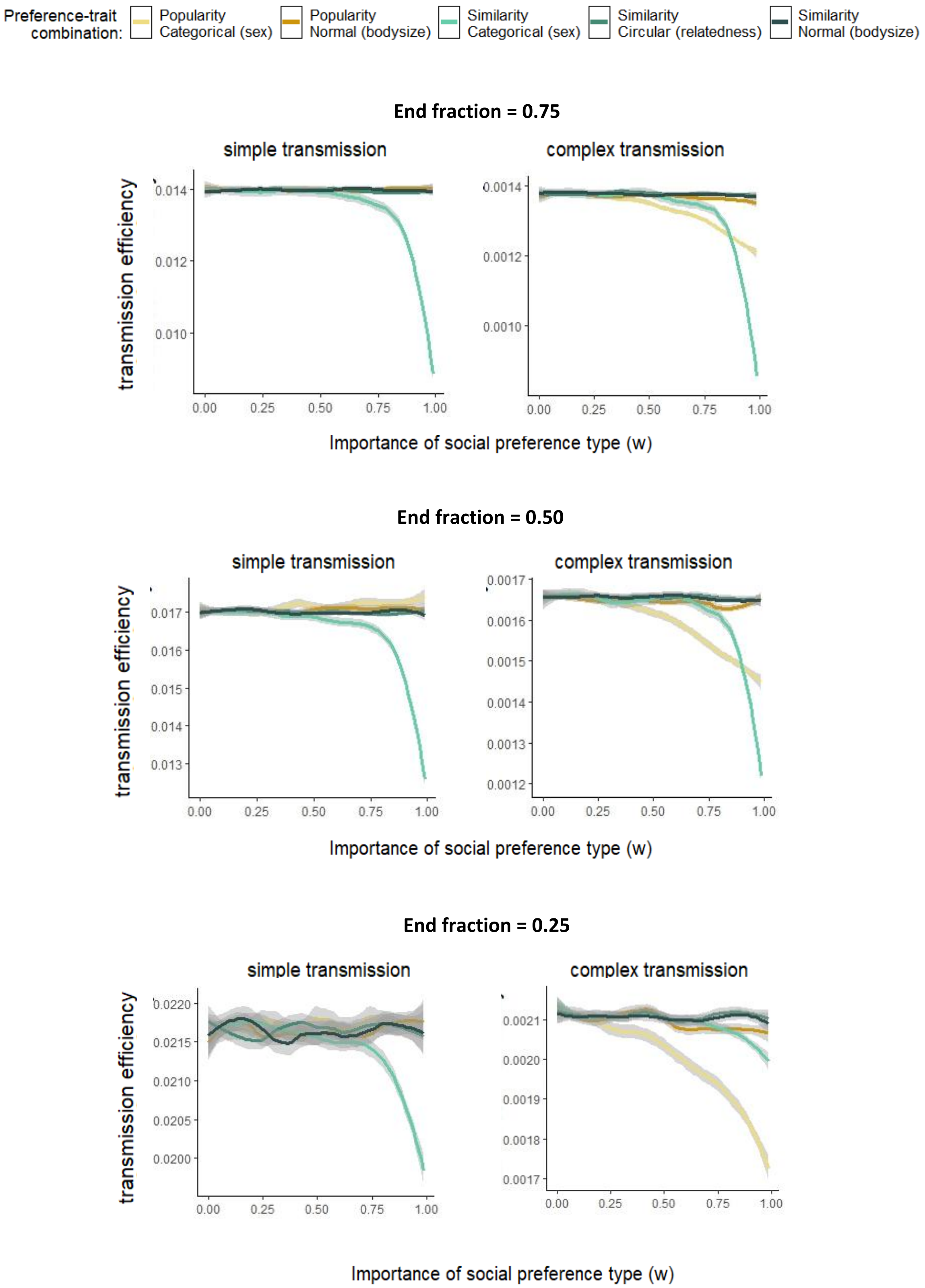

Fig. S8. Effects of trait-based social preferences on social network transmission, for different end fractions. The change in transmission efficiency with increased importance of social preferences, for each preference-trait combination (see legend) and two types of (unweighted) transmission (given by subplot headers), for different end fractions (fraction of individuals that have been infected by the end of the simulation, given by row headers).

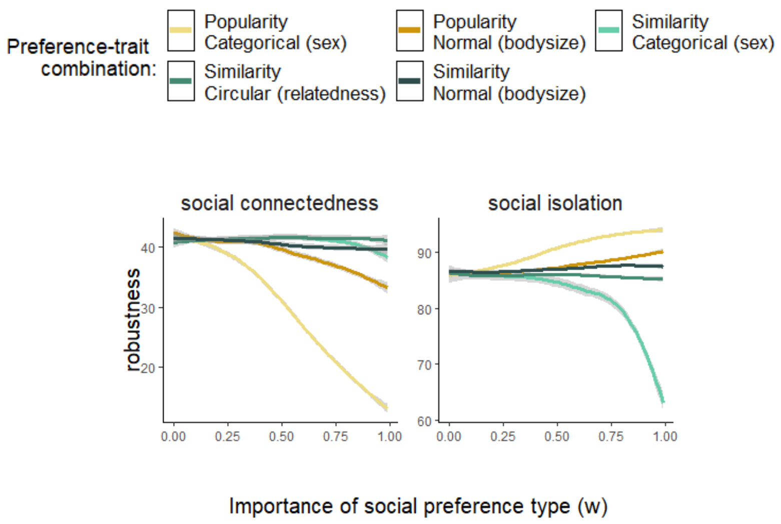

Fig. S9. Effects of trait-based social preferences on social network robustness, for removal based on weighted network metrics. The change in robustness with increased importance of social preferences, for each preference-trait combination (see legend), for two types of loss of individuals (network nodes; given by subplot headers), where the node removal is based on weighted metrics (high and low weighted degree, respectively).

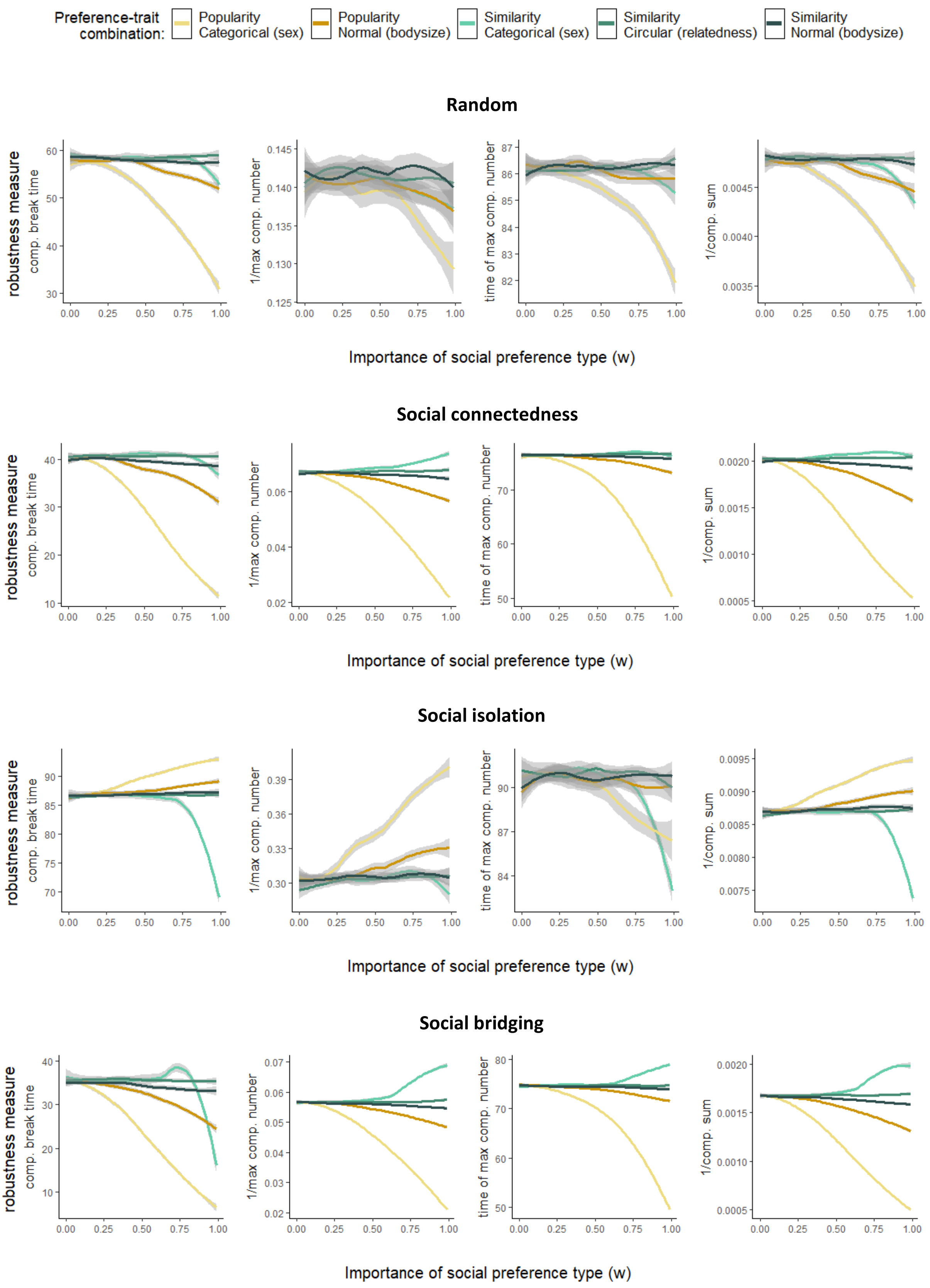

Fig. S10. Effects of trait-based social preferences on social network robustness, for all robustness measures. The change in robustness with increased importance of social preferences, for each preference-trait combination (see legend), for four types of robustness measure (comp. = component. Left column = robustness measure used in main analysis). Each row is for a different node removal type (given by row headers).
