# Supplementary material for: Far-reaching consequences of trait preferences for animal social network structure and function
Source: Behav Ecol. 2025 Nov 21;37(1):araf132. doi: 10.1093/beheco/araf132 (PMC12784201; doi:10.1093/beheco/araf132)
Supplement: araf132_Supplementary_Data [file araf132_supplementary_data.zip › Version for press, Suppl Mat A-D, trait prefs.pdf]

Supplementary Material

Brask *et al.*: Far-reaching consequences of trait preferences for animal social network structure and function

Contents

Supplementary Material A: Mathematical description of the trait preference model

Supplementary Material B: Using the model for analyzing empirical networks

Supplementary Material C: Mathematical method descriptions

Supplementary Material D: Mechanisms behind the network function results

A. Mathematical description of the trait preference model

Here we describe the general trait preference model, where the traits and preference functions are not specified. An example of a version of the model with specified traits and preference function equations can be found in the model specification for the simulation study (Suppl. Mat. C).

The model can be described mathematically as follows:

$$S(i,j) = \sum_{b \in \boldsymbol{B}} (w_b * S_b(i,j)) + w_{rand} * S_{rand}(i,j) \ , \tag{A1}$$

where

$$S_b(i,j) = f_b\big(V_b(i), V_b(j)\big) \ . \tag{A2}$$

The *social attraction* S between two individuals i and j (Eq. A1) depends on their *trait-specific social attractions* S_b (for each trait b in the set of traits *B*), and their *random social attraction* S_{rand}. The amount of influence each trait (and the preference it is combined with) has on the social attraction is given by its weight w_b, and the amount of influence of randomness is given by its weight w_{rand}. The trait-specific social attraction S_b (Eq. A2) for any given trait b is determined by the two individuals' values of that trait (V_b), and a *preference function* (f_b) that transforms the trait values into the trait-specific social attraction value, and thereby describes the way in which the trait influences the social attraction (similarly to linking functions in hidden variable models, e.g. Caldarelli et al. 2002, Servedio et al. 2004).

For similarity preferences, the preference function must transform the trait values to a distance (or closeness) measure (i.e. the social attraction value is relative the distance between the two trait values), whereas for popularity, they must transform them to a magnitude measure. Apart from that, the functions may take on different forms within each preference type. Different traits may be combined with identical or different preference functions in any given edition of the model.

Given Eq. A2, the weight w_b (Eq. A1) for a given trait can be seen both as the importance of the trait, and as the importance of the preference type that the trait is used with.

References for Supplementary Material A

Caldarelli G, Capocci A, De Los Rios P & Munoz MA. 2002. Scale-free networks from varying vertex intrinsic fitness. Physical review letters, 89(25), 258702

Servedio VD, Caldarelli G & Buttà P. 2004. Vertex intrinsic fitness: How to produce arbitrary scale-free networks. Physical Review E—Statistical, Nonlinear, and Soft Matter Physics, 70(5), 056126

B. Using the model for analyzing empirical networks

Here we demonstrate the idea that the trait preference model can be used for analyzing observed networks. The approach is to estimate the importance (the weight parameters w, Eq. A1 in Suppl. Mat. A) of each possible preference-trait combination, where the combinations are made from combining each observed (measured) trait with each preference type (similarity and popularity). This could be used to statistically infer which traits are important for the observed network structure, and via which preference type they are important. The parameterized model could also be used to generate simulated networks based on the real network. For the parameterization, we combine the model with a Bayesian estimation approach (see below for details of the parameter estimation procedure).

We note that if one or more preference-trait combinations are found to be important (significant) for a given real network, this could be due to both active and passive preferences (see main text). Thus, inference with the model provides information on which traits are important for the network structure, and whether the structure connected to that trait corresponds to similarity or popularity patterns (trait-based assortment or correlations between trait values and connectedness, respectively, see main text). Assumed active preferences (active trait-based social partner choice) may then be confirmed by system-specific knowledge or experiments.

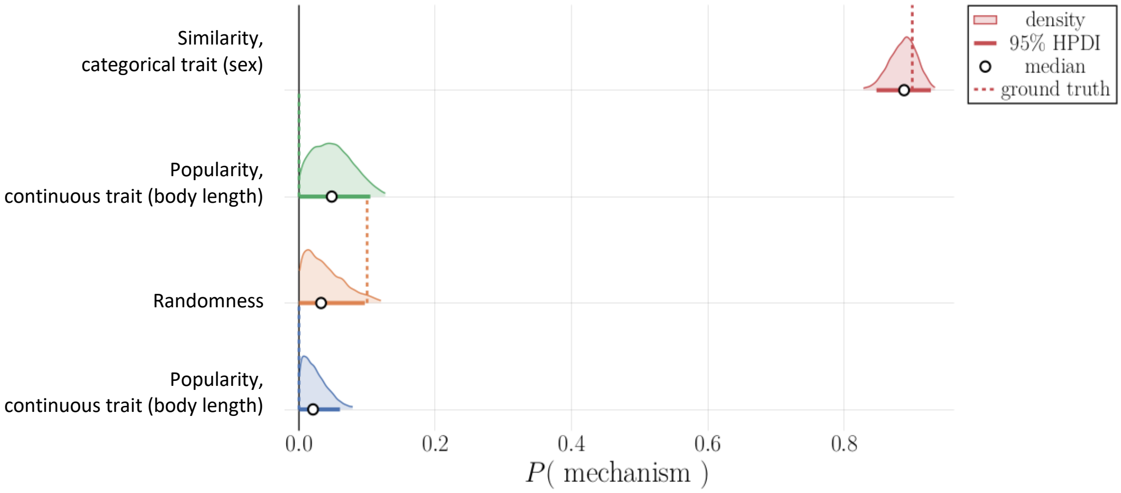

Fig. B1. Inferring the importance of different preference-trait combinations for an observed network. The figure shows the parameter distributions obtained from the parameterization procedure for each combination of preference (similarity or popularity) and trait (those that have been measured, here sex and body length). Distributions that do not overlap with zero can be considered to indicate statistical significance. Dotted lines indicate the ground truth (parameters as they were set in the model when generating the 'observed' network to which the parameter estimation was applied). Thus, in this example the result suggests that similarity based on sex is playing an important role in the 'observed' network, which fits with the ground truth.

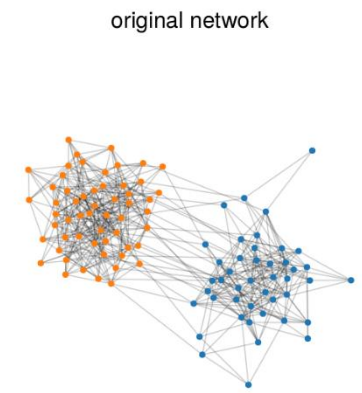

Fig. B2. Generating networks based on a trait preference model parameterized by an observed network. The 'observed' (original) network is shown together with a network generated by the parameterized model (based on parameters sampled from the parameter distributions). The network node colors correspond to the two categories of the categorical trait (sex) that was found to be of importance in the original network.

To parameterize the model with real data, we would need an observed network and data from the individuals on one or more traits. For the example presented here, we use simulated data where the ground truth is known; the 'observed' network here is thus generated with the model, based on set parameters and simulated trait values.

The Bayesian estimation procedure outputs an inferred parameter distribution for each preference-trait combination (Fig. B1). We find that the inference overall fits with the ground truth, with similarity based on a categorical trait (sex) being estimated as being of high importance for the network, and the other preference-trait combinations being estimated as being of low importance, which corresponds to the settings we used to generate the 'observed' network. Using the parameterized model, we can then generate networks that are very similar to the 'observed' network (Fig. B2).

Importantly, estimation from real data carries inherent challenges, and while we have here demonstrated the idea using simulated 'observed' data, methodology for actual real data needs to be thoroughly tested and refined before it may be used as a parameterization and inference tool in scientific studies.

Methods for estimating model parameters

We use an inference approach that provides a relatively simple and direct way of estimating parameters while respecting the model and parameter interpretation given in the model presentation (the main text and Suppl. Mat. A and C). We calculate the trait-specific social attractions (S_b in Eq. A1

in Suppl. Mat. A) with the same preference functions as defined for the model specification used in the simulation study (Eq. C1-C7 in Suppl. Mat. C), using observed trait values and a value of 0.5 across all edges for randomness. We normalize the overall social attraction S such that the mean corresponds to the density of the observed network. This gives us the probability $P(i,j)$ of an edge between individuals i and j. To infer the unknown parameters, we choose an uninformative Dirichlet distribution as prior, which ensures that the values sum up to one. Finally, we use a Bernoulli likelihood to compare observed edges with the calculated probabilities $P(i,j)$. The statistical model then reads:

$$w_b \sim Dirichlet(1) \tag{B1}$$

$$S(i,j) = \sum_b w_b S_b(i,j) \ \ \forall i-j \tag{B2}$$

$$P(i,j) = S(i,j)\ \frac{\underline{P}}{\underline{S}} \ \ \forall i,j \tag{B3}$$

$$\underline{S} = \frac{1}{N_d} \sum_{i,j} S(i,j) \tag{B4}$$

$$e(i,j) \sim Bernoulli\big(P(i,j)\big)\ , \tag{B5}$$

where b Is a social preference mechanism with a specific trait, $\underline{P}$ is the observed network density, N_d is the number of dyads, and $e(i,j)$ indicates if an edge between node i and j exists in the empirical network. We implement the inference algorithm in the Julia programming language (Bezanson et al. 2017) using the Turing.jl package (Ge et al. 2018) for Bayesian inference. Specifically, we estimate the parameters using a No-U-Turn sampler (Hoffman & Gelman 2014), i.e. a variant of the Hamilton Monte-Carlo sampler with a target acceptance rate of 0.9 and a maximum tree-depth of 5. We run 6 parallel chains with 2000 samples each and discard the first 1000 for warm-up. All inference results report no divergent transitions. Also, the maximum Gelman–Rubin diagnostic and R-hat statistics are below 1.1 for all simulations, thus indicating sufficient mixing and convergence of the Monte-Carlo chains.

References for Supplementary Material B

Bezanson J, Edelman A, Karpinski S & Shah VB. 2017. Julia: A fresh approach to numerical computing. SIAM review 59(1), 65–98

Ge H, Xu K & Ghahramani Z. 2018. Turing: a language for flexible probabilistic inference. In: International Conference on Artificial Intelligence and Statistics, AISTATS 2018, pp. 1682–1690

Hoffman MD & Gelman A. 2014. The No-U-Turn sampler: adaptively setting path lengths in Hamiltonian Monte Carlo. J. Mach. Learn. Res., 15(1), 1593-1623

C. Mathematical method descriptions

Generating networks based on trait preferences

Mathematical model specification

The version of the trait preference model that we use for the simulation study involves two traits, which we refer to as the *similarity trait* and the *popularity trait* (as they are used with similarity and popularity preferences, respectively). For this model version, the social attraction S between individuals i and j is given by

$$S(i,j) = w_{pop} * S_{pop}(i,j) + w_{sim} * S_{sim}(i,j) + w_{rand} * S_{rand}(i,j) \quad . \qquad \text{(C1)}$$

Here, S_{pop}, S_{sim}, and S_{rand} are partial social attractions based on the popularity trait, the similarity trait, and randomness, and w_{pop}, w_{sim}, and w_{rand} are their respective weights (giving the importance of each trait, and hence also of each preference type, for the social attraction $S(i,j)$). The weights w all lie in the interval [0,1] and must sum to 1, and S_{rand} is drawn uniformly at random from the interval [0,1]. Together with the condition that S_{pop}, S_{sim} and S_{rand} also each lies in the interval [0,1], this ensures that the social attraction S lies between 0 and 1. The trait-specific social attractions are given by the following preference functions:

$$S_{pop}(i,j) = \frac{mag(i,j)}{max\,(mag(dyad))} \qquad \text{(C2)}$$

and

$$S_{sim}(i,j) = 1 - \frac{dist(i,j)}{\max(dist(dyad))} \quad , \qquad \text{(C3)}$$

where $mag(i,j)$ and $dist(i,j)$ are, respectively, the joint magnitude of the trait values of i and j, and the distance between the trait values of i and j (details below), with max taking the maximum of these measures across all dyads. Hence, here higher values of the popularity trait are more popular, and individuals prefer others that have similarity trait values closer to their own. The denominators ensure that the social attractions are between 0 and 1.

It is necessary to specify the joint trait value magnitude (mag) and distance ($dist$) separately for continuous and categorical traits. For continuous traits the joint magnitude and distance are given by:

$$mag(i,j) = V_{pop}(i) + V_{pop}(j) \tag{C4}$$

and

$$dist(i,j) = |V_{sim}(i) - V_{sim}(j)| \ , \tag{C5}$$

where V_{pop} and V_{sim} are popularity and similarity trait values, respectively. For categorical traits, the measures are given by:

$$mag(i,j) = \begin{cases} 1 & if\ V_{pop}(i)\ and\ V_{pop}(j) = the\ popular\ trait\ category \\ 0.5 & if\ V_{pop}(i)\ or\ V_{pop}(j) = the\ popular\ trait\ category \\ 0 & otherwise \end{cases} \tag{C6}$$

and

$$dist(i,j) = \begin{cases} 0 & if\ V_{sim}(i) = V_{sim}(j) \\ 1 & otherwise \end{cases} \tag{C7}$$

Specification of trait type distributions

Categorical, corresponding to sex: trait values are on a categorical scale and are drawn with equal probability from each category. We use two categories for the main analysis, and we also investigate the effect of more categories.

Continuous normal, corresponding to body size: trait values are on a continuous scale with a maximum and minimum, and are drawn from a truncated normal distribution, with max = 1, min = 0, mean = 0.5, SD = 0.25.

Continuous circular, corresponding to genetics: trait values are on a continuous, circular scale and are drawn from a uniform distribution. The scale has a circumference of 2 (i.e. the distance between values ranges from 0 to 1).

Importance values

We vary the weights w_{sim} and w_{pop} (the importance of similarity and popularity preferences, Eq. C1) across the range [0, 0.99] in steps of 0.01 (we do not include 1 because single-component networks cannot be generated when w_{sim} = 1) and generate an ensemble of networks for each w_{sim}, w_{pop} combination where the condition that their sum cannot exceed 1 is fulfilled (note that w_{rand} is given

by the condition w_{sim}+ w_{pop}+ w_{rand} = 1). We do this for different types of traits, as explained in the main text. We then quantify average structural and functional aspects of each network ensemble and study these ensemble averages as a function of w_{sim} and w_{pop}.

Edge drawing procedure

To determine the positions of edges, we use a procedure that corresponds to that of the G(n,L) model for Poisson networks (Erdős & Rényi 1959) but depends on the dyadic social attractions: We draw a weighted random sample of L dyads, where the probability of being drawn is proportional to the social attraction $S(i,j)$, and L is the number of links to be placed in the network (given by $L = \bar{k} * n/2$, where $\bar{k}$ is a specified average degree and n is a specified network size, i.e. number of nodes), and we then place an edge between the two nodes of all the drawn dyads. We create unweighted networks by giving each present edge an edge weight of 1, and we create weighted networks by giving each present edge an edge weight drawn from a normal distribution with mean = $S(i,j)$ and sd = 0.05 (and with any drawn values < 0 set to 0.001 and any drawn values >1 set to 1).

Measuring structural and functional properties of the generated networks

Calculation of network metrics

We use both an unweighted and a weighted version of each network metric.

Degree variation: Calculated as the variance in the unweighted or weighted degree.

Degree assortativity: Calculated as Newman assortativity for continuous variables (Newman 2002; Newman 2003), with connectedness measured as unweighted or weighted degree.

Clustering: Calculated as the ratio of closed triangles in the network divided by the number of connected triples (for the unweighted version), or according to Barrat et al. (2004) (for the weighted version).

Mean distance: the average shortest path length between dyads, calculated as the minimum number of edges between them (for the unweighted version) or the minimum path weight between them (for the weighted version). Given that individuals connected through stronger social links are socially closer, we calculate the path weight for a path ρ between i and j as

$$\Lambda_{i,j,\rho} = \sum_{a \in A_{i,j}} \frac{1}{\Phi_a} , \quad \text{(C8)}$$

where Φ_a is the edge weight of the edge a, and A is the set of edges on the path.

Popularity trait – degree correlation: calculated as the Pearson correlation between the individuals' popularity trait values and their unweighted degrees.

Similarity trait assortativity: calculated as Newman assortativity (Newman 2002; Newman 2003) for categorical or continuous variables (depending on the distribution of the similarity trait).

Transmission models

Simple transmission: We use the SI model to model simple transmission. When edge weights are taken into account, the probability of being infected for a node i at time t is given by

$$p_{i,t} = 1 - \prod_{j \in m_{i,t}} \left(1 - \beta * \Phi_{i,j}\right) \ , \tag{C9}$$

where β is the transmission probability (set to 0.01 in our simulations), $\Phi_{i,j}$ is the weight of the edge between i and j, and $m_{i,t}$ is the set of neighbours of i that are infected at time t. For the case where edge weights are not influencing transmission (i.e. for binary networks), this becomes equivalent to

$$p_{i,t} = 1 - (1 - \beta)^{\mu_{i,t}} \ , \tag{C10}$$

where μ is the number of neighbours infected at time t.

Complex transmission: We use a proportional transmission model to model complex transmission. When edge weights are taken into account, the probability of being infected for a node i at time t is given by:

$$p_{i,t} = \frac{\sum_{j \in m_{i,t}} \Phi_{i,j}}{\sum_{j \in M_i} \Phi_{i,j}} \ * \ \gamma \ , \tag{C11}$$

where $\Phi_{i,j}$ is the weight of the edge between i and j, $m_{i,t}$ is the set of neighbours of i that are infected at time t, M_i is set of all neighbours of i, and γ is a transmission parameter (set to 0.01 in our simulations). For the case where edge weights are not affecting transmission, this fraction is equivalent to the proportion of neighbors that are infected at time t.

Robustness measures

To study different dimensions of network robustness, we quantify the following three additional measures of robustness (each timestep corresponds to a node removal, and a component is a network part that is disconnected from other network parts):

Inverse max component number: The inverse of the maximum number of network components reached during the node removal process, i.e. the maximum level of fragmentation the network reaches when individuals are lost from the population.

Max component number time: The time at which the maximum number of network components is first reached, i.e. the number of individuals that are lost before the network reaches maximum fragmentation.

Inverse summed component number: The inverse of the number of network components summed over the full time-range, i.e. the extent to which the network breaks into pieces when individuals are lost from the population.

References for Supplementary Material C

Barrat A, Barthelemy M, Pastor-Satorras R & Vespignani A. 2004. The architecture of complex weighted networks. Proceedings of the national academy of sciences, 101(11), 3747-3752

Erdős P & Rényi A. 1959. On Random Graphs. I. Publicationes Mathematicae. 6: 290–297

Newman MEJ. 2002. Assortative mixing in networks, Phys. Rev. Lett. 89, 208701

Newman MEJ. 2003. Mixing patterns in networks, Phys. Rev. E 67, 026126

D: Mechanisms behind the network function results

Here we describe likely mechanisms behind different aspects of the transmission and robustness results.

Transmission

Trait preferences leading to decreases in transmission efficiency

The mechanisms behind the decrease in transmission efficiency (Fig. 3A) depends on the preference-trait combination. The strong decrease for similarity preferences with a categorical trait under high preference importance may be explained by increased network modularity (Fig. 1A), with strong increases in clustering and mean distance (Fig. 1C and Fig. 2). The considerable decreases in efficiency for the networks based on popularity preferences may be explained by increased network centralization (Fig. 1A), with strong increases in the degree measures (Fig. 1C and Fig. 2).

Different results for simple and complex transmission

For simple transmission, popularity with a categorical trait has the strongest effect on transmission efficiency, whereas for complex transmission, similarity with a categorical trait has the strongest effect (Fig. 3A). This pattern may be explained by the following: The network based on popularity with a categorical trait is characterized by increased degree variation and negative degree assortativity (Fig. 1), and a centralized structure where nodes with the popular trait value are central and nodes with the unpopular trait value are peripheral. Under simple transmission (where a node's infection chance is relative to its number of infected neighbours), the peripheral nodes are hard to reach for the infection because of their low number of neighbours, leading to the strong effect on transmission efficiency for this type of network. Under complex transmission (where a node's infection chance is relative to its *proportion* of infected neighbours), these nodes do not pose the same challenge for the transmission because their low degree means that only few neighbours need to be infected to give a high proportion of infected neighbours. Hence, under complex transmission this type of network does not lead to the same strong effect on transmission relative to the other types of networks, and the preference-trait combination having the strongest effect on transmission therefore differs between the two transmission types.

Robustness

Trait preferences leading to decreases in robustness

The strong decreases in robustness for popularity with a categorical trait (as well as the smaller decreases for popularity preferences with the 'normal' trait) across most types of node loss (Fig. 3B) may be explained by the increased degree variation (Fig. 1C), which creates hubs (highly connected nodes) that are more likely to lead to network fragmentation when they are lost. This effect is not seen under 'social isolation' node loss, because here the lowest-degree nodes are targeted, which have more peripheral positions in these networks. The strong non-linear effect with sudden downturns for similarity with a categorical trait fits with the nonlinear effect of this preference-trait combination on network metrics (particularly mean distance; Fig. 1C).

Robustness results for random and social connectedness node loss

Random node loss and node loss based on social connectedness show quite similar robustness results (Fig. 3B), although the robustness is overall lower for loss based on social connectedness. The lower robustness for social connectedness is expected, as removing the more connected nodes should fragment the networks quicker. The fact that the preferences do not lead to long-tailed degree distributions (Fig. S6) acts as a counterweight to this effect and means that the networks do not break down right away.

Different direction of effect of popularity preferences under social isolation node loss

The popularity preferences lead to increased robustness against social isolation node loss, whereas they lead to decreased robustness against the other types of node loss (Fig. 3B). This may be explained by the fact that networks based on popularity preferences have increased core-periphery structure and increased degree variation (Fig. 1). This increases the robustness for these networks against social isolation node loss, as the targeted nodes in this case are peripheral in the networks and their loss is therefore unlikely to cause network fragmentation.
